# Hypoglycemic Activity of *Tilia americana*, *Borago officinalis*, *Chenopodium nuttalliae*, and *Piper sanctum* on Wistar Rats

**DOI:** 10.1155/2019/7836820

**Published:** 2019-04-16

**Authors:** Martha P. Rodríguez-Magaña, Paula Cordero-Pérez, Catalina Rivas-Morales, María A. Oranday-Cárdenas, Diana P. Moreno-Peña, David G. García-Hernández, Catalina Leos-Rivas

**Affiliations:** ^1^Facultad de Ciencias Biológicas, Departamento de Química, Universidad Autónoma de Nuevo León, Mexico; ^2^Facultad de Medicina, Departamento de Medicina Interna, Unidad de Hígado, Universidad Autónoma de Nuevo León, Mexico

## Abstract

Diabetes mellitus (DM) is considered the epidemic of the 21st century. Traditional medicine uses plants to treat DM; many of these have hypoglycemic effects in both animal models and diabetic patients. Our objective was to evaluate the hypoglycemic activity of *Tilia americana*, *Borago officinalis*, *Chenopodium nuttalliae*, and *Piper sanctum* on diabetic rats. The methanolic extracts of the plants under study were obtained by Soxhlet extraction. Toxicity was evaluated on *Artemia salina*; the antioxidant potential was evaluated using the DPPH technique. Hypoglycemic capacity at doses of 250 and 500 mg/kg was tested on Wistar rats with diabetes induced by alloxan (120 mg/kg). The toxicity on *A. salina* was null for the extracts of *B. officinalis* and *P. sanctum*, moderate for *T. americana*, and highly toxic for *C. nuttalliae*. The relevant extract of *T. americana* var. mexicana showed antioxidant activity. Three plants of the studied plants showed hypoglycemic activity: *Tilia Americana* (*p* = 0.0142), *Borago officinalis* (*p* = 0.0112), and *Piper sanctum* (*p* = 0.0078); *P. sanctum* was the one that showed the greatest reduction in glucose levels at a lower dose.

## 1. Introduction

Diabetes mellitus (DM) is a metabolic disease characterized by an elevated level of blood glucose that is associated in the long term with the dysfunction of different organs. Left untreated, it can cause blindness, renal insufficiency, myocardial infarction, cerebrovascular accidents, or amputation of the lower limbs [1].

DM is considered the epidemic of the 21st century, and its prevalence has increased in low- and middle-income countries. It is estimated that DM caused 1.6 million deaths in 2015 (WHO 2017) and is projected to be the seventh cause of mortality by 2030 [2, 3].

Most diabetic patients are included into two groups, type I (total deficiency of insulin secretion) and type II (insulin resistance). Treatment of type I diabetes is based on diet and the administration of insulin. Treatment of type II diabetes starts with diet and physical exercise; if the response is not adequate, then hypoglycemic drugs are administered orally (sulfonylureas and metformin).

Many plants used in traditional medicine (*Momordica charantia*, *Terminalia paniculata* Bar, *Scrophularia ningpoensis*, and *Anemarrhena asphodeloides*, among others) are used to treat patients with DM in various parts of the world, mainly in developing countries. The hypoglycemic effect of a large number of these plants or their preparations has been confirmed in animal models [4, 5], as well as in diabetic patients [6–8]. Among the active compounds with hypoglycemic effect that have been isolated are alkaloids, terpenoids [9], flavonoids [10], and saponins [11].

In this study, four plants of different families used in traditional medicine were chosen to evaluate hypoglycemic activity.

The leaves of *Piper sanctum* (Piperaceae) are used as tea to treat stomach cramps, coughs, bronchitis, tuberculosis, asthma, and colds and as a poultice in skin irritations and arthritis. The presence of alkaloids and lactones with antibacterial activity is reported for this plant [12]. Another species, *P. betle*, shows the presence of terpenes and phenols with relevant antidiabetic activity [13].

*Tilia americana* var. mexicana (Malvaceae) has anxiolytic and sedative properties as well as antioxidant activity and is used as a soother or relaxant [14] for the neural damage induced by intestinal ischemia [15]. *T. cordata* is used in traditional medicine for the treatment of diabetes, and other plants of the same family have hypoglycemic activity [16].

Oil of *Borago officinalis* (Boraginaceae) is marketed as a nutritional supplement for being a source of oleic and linoleic acids. Borage seed oil is used in the treatment of various diseases such as multiple sclerosis, diabetes, heart disease, arthritis and eczema [17], and gastrointestinal and cardiovascular disorders as well as for atopic dermatitis [18]. This plant has anti-inflammatory and antioxidant properties due to the flavonoids and sterols that have been isolated from it [19, 20]. Another plant from the same family, *Heliotropium strigosum*, reduced the blood glucose level of diabetic mice induced by alloxan, and the presence of alkaloids, tannins, proteins, steroids, and flavonoids is reported [21].

*Chenopodium nuttalliae* is used to combat mental and physical fatigue. To date, there is no activity described for *C. nuttalliae* (Chenopodiaceae); however, several plants of this family show hypoglycemic activity [22]. Other plants of the same genus, such as *C. ambrosioides*, show antifungal, antiaflatoxigenic, and antioxidant activities [23].

Due to the aforementioned background, our objective was to evaluate the hypoglycemic activity of *T. americana*, *B. officinalis*, *C. nuttalliae*, and *P. sanctum* on diabetic rats.

## 2. Materials and Methods

### 2.1. Vegetal Material

*B. officinalis* was collected from Saltillo, Coahuila (25.4383° N, 100.9737° W) (record number 27344); *C. nuttalliae* from Monterrey, Nuevo Leon (25.6866° N, 100.3161° W) (record number 27799); *T. americana* var. mexicana from Morelia, Michoacan (19.7060° N, 101.1950° W) (record number 27797); and *P. sanctum* from Mexico City (19.4326° N, 99.1332° W) (record number 27798). The plants were identified in the Department of Botany of the College of Biological Sciences, Universidad Autónoma de Nuevo León (UANL), Mexico.

The material was dried in the shade at room temperature, and the leaves were separated and crushed in a manual mill. For each plant, 200 g were taken and deposited in a Soxhlet extractor with methanol as solvent. The samples were left in reflux for 40 h to later concentrate the extract with a Büchi-type rotary evaporator at a temperature lower than 60°C. The concentrate was dried at ambient temperature and was stored in amber-colored vials at 4°C until use. For administration in the animal model, the dry extracts were solubilized in physiological solution using a sonicator.

### 2.2. Phytochemical Profile of Extracts

The methanolic extracts were subjected to a phytochemical profile by means of qualitative colored tests for the identification of flavonoids (Shinoda test), coumarins (NaOH test), alkaloids (Dragendorff test), sugars (Antrona test), sesquiterpenectones (Baljet test), tannins (FeCl_3_ test), sterols, and terpenoids (Liebermann-Burchard test) [24].

### 2.3. Lethality Test on *Artemia salina*

Artificial seawater (Instant Ocean) was prepared, adjusting the pH to 7.8. The aquarium was ventilated with a pump for 24 h. *A. salina* nauplii were hatched in a rectangular glass container (17 cm × 14 cm × 7 cm) with a dark chamber for cyst incubation and illuminated to obtain the nauplii by phototropism. They were incubated 48 h at room temperature (23-25°C) with constant aeration, after which the assay was carried out in 96-well microplates (Costar, Corning, NY, USA). Ten nauplii were deposited in wells containing a final volume of 200 *μ*L and concentrations of 100-1000 *μ*g/mL of each extract of the plants under study and incubated for 24 h, and dead nauplii were counted using a stereoscope [25].

### 2.4. Experimental Animal Model

This study was carried out under the approval of the ethics committee of the College of Medicine of the UANL (registration no. HI11-002) following the provisions of the Official Mexican Standard NOM-062-ZOO-1999 technical specifications for the production, care, and use of laboratory animals. The animals were kept under appropriate conditions of light, temperature, and humidity, and they were given standard diet for rodents and water *ad libitum*.

### 2.5. Diabetes Animal Model

Diabetes was induced in male Wistar rats (180-240 g) by the administration of a single dose of alloxan, intraperitoneally (120 mg/kg of weight). After injection, they were provided with a 5% dextrose solution in the drinking fountains to overcome the hypoglycemic phase. After 48 h, blood samples were collected through a puncture at the tip of the tail and the glucose levels were determined by test strips using a glucometer (ACCU CHECK Roche); if the level was greater than 250 mg/dL, the rat was selected for the study [26].

### 2.6. Experimental Design

We used 72 diabetic rats, which were distributed randomly into 8 groups with 9 rats each. The basal glucose was measured in the test animals, and subsequently, a methanolic extract of each of the plants at a dose of 250 and 500 mg/kg of weight or physiological solution (control group) was administered by means of an intragastric probe. After 4 h, a second glucose measurement was made. These doses were determined based on a review of the antidiabetic activity of plants made by our group, in which reported doses oscillated between 200 and 500 mg/kg [27].

### 2.7. Antioxidant Activity

To evaluate the antioxidant activity, the method of reduction of the 1,1-diphenyl-2-picrylhydrazyl (DPPH) radical was used [28]. The methanolic extracts were evaluated at concentrations of 3.125 to 1000 ppm. The DPPH (Sigma-Aldrich) was prepared to 125 *μ*M in methanol, 100 *μ*L of each sample was taken, and 100 *μ*L of DPPH was added; the samples were allowed to stand for 30 min protected from light. The absorbance at 517 nm was measured using a spectrophotometer (Jenway 320d). As a positive control, a solution of tocopherol (Sigma-Aldrich) was used and as negative control EtOH; the reduction percentage was calculated using

(1) $$\begin{array}{c} \%\text{Reduction}=\frac{\text{Absorbancy negative control}–\text{Absorbancy sample}}{\text{Absorbancy negative control}}\times 100. \end{array}$$

### 2.8. Statistics

The mean lethal dose (LD_50_) and the mean lethal concentration (LC_50_) were determined by means of a probit regression with the software SPSS version 17. In the animal model, a nonparametric “*t*” test was performed to determine the significant difference in the reduction of glucose for each group using the GraphPad 7.0 program; a value of *p* < 0.05 was considered significant.

## 3. Results and Discussion

### 3.1. Results

The phytochemical profile of the four plant extracts showed flavonoids, sugars, sterols, and terpenoids. *B. officinalis* also showed quinones, sesquiterpenlactone, alkaloids, and tannins, while in *P. sanctum*, sesquiterpenlactone, coumarins, alkaloids, and tannins. The extracts of *T. americana* showed coumarins and tannins, and those of *C. nuttalliae* also showed coumarins.

#### 3.1.1. Toxicity Test on *Artemia salina*

We evaluated the toxic activity of the methanolic extracts of *T. americana* var. mexicana, *B. officinalis*, *C. nuttalliae*, and *P. sanctum* against the nauplii of *A. salina*. The extracts with lower toxicity were *B. officinalis* and *P. sanctum* (Table 1). The toxic activity was calculated according to the classification reported by Syahmi et al. [29] based on their LC_50_ value (0-100 *μ*g/mL, high; 100-500 *μ*g/mL, moderate; 500-1000 *μ*g/mL, low; and a value higher than 1000 *μ*g/mL, nontoxic).

#### 3.1.2. Hypoglycemic Activity

**Figure 1:**
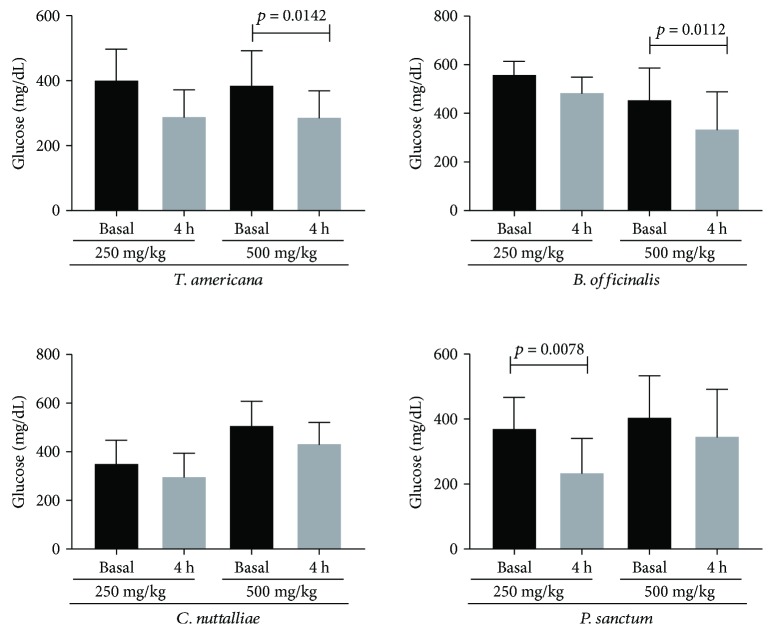
Glucose levels, basal and 4 hours after the administration of the extracts under study at doses of 250 and 500 mg/kg. Values are expressed as mean ± SD.

The group of diabetic rats maintained their elevated glucose levels during the study period (baseline, 380.6 ± 68.1 mg/dL; 4 h, 377.88 ± 70.9 mg/dL; *p* > 0.05); the negative control group maintained its normal glucose levels (baseline, 116.0 ± 15.4 mg/dL; 4 h, 121.3 ± 12.5 mg/dL; *p* > 0.05). When evaluating the two doses (250 and 500 mg/kg of weight) of the methanolic extracts of each plant, only *B. officinalis and T. americana* showed relevant hypoglycemic activity 4 h posttreatment at a dose of 500 mg/kg (*p* = 0.0112), whereas *P. sanctum* higher showed antihypoglycemic effect at a dose of 250 mg/kg (*p* = 0.0078) of weight (Figure 1).

#### 3.1.3. Antioxidant Activity

When evaluating the antioxidant activity of the methanolic extracts (DPPH), it was observed that the extract of *T. americana* showed a higher activity compared with the other extracts under study with an EC_50_ of 8.84 ± 1.05 mg/mL (Table 2).

### 3.2. Discussion

The interest in natural alternatives for the treatment of diabetes has led to the evaluation of a large number of plants in the search for bioactive compounds with possible hypoglycemic activity; however, it is important to rule out a toxic effect of plants when evaluating said activity. In the rat model, our study plants showed hypoglycemic activity.

We found that *T. americana* showed hypoglycemic activity at the dose of 500 mg, although it presented moderate toxicity. No previous studies showed such activity for this plant, although this activity is reported in another species of the same genus [15]. In this study, this plant was the one that showed the greatest antioxidant activity, even greater than the control (Table 2). This activity may be related to the presence of flavonoids, previously reported by other authors [14, 30]. *T. americana* was moderately toxic on *A. salina*, which, to our knowledge, has not been previously reported.

Several plants of the family Chenopodiaceae have medicinal effects. For example, *Anabasis articulata* has antihyperglycemic effects [31]; *Salsola kali*, *S. soda*, and *S. oppositifolia* are inhibitors of alpha amylase [32]; and *Atriplex halimus* is an antidiabetic and *Chenopodium ambrosioides* is hypoglycemic [33]. In this study, we found that *C. nuttalliae* showed low hypoglycemic activity. Chikhi et al. [22] report a significant antioxidant for this plant, whereas we found low antioxidant activity. *C. nuttalliae* was highly toxic for *A. salina*, which has not been previously reported; 2,873.23 mg of 100·g^−1^ dry matter of saponins was found in this plant's sprouts, conferring moderate toxicity [33].

*B. officinalis* showed hypoglycemic activity, and this is increased with respect to the dose used. It has been described that this plant is rich in gamma-linolenic acid that has been used as a medicine to treat various diseases such as diabetes, local eczema, heart disease, cyclical mastalgia, arthritis, and multiple sclerosis [34, 35]. In another plant of the *B. officinalis* family, this activity has also been reported [21]. The antioxidant activity found was low, and although the phytochemical profile showed the presence of flavonoids, to which this property is attributed, it also contains other compounds which can interfere in biological activity [19, 20, 36, 37]. It showed no toxicity on *A. salina*, and there are no reports of toxicity in this plant.

The Piper species show different activities; for example, *P. auritum* has terpenes, flavonoids, essential oils, and alkaloids and *Piper betle* has terpenes and phenols that are related to hypoglycemic [13, 38] and antioxidant activity [39], whereas for *P. sanctum*, only antibacterial activity related to the presence of alkaloids and lactones has been reported [12]. In this study, we found sterols, flavonoids, coumarins, alkaloids, and tannins, which may be related to biological activity. Of the four plants studied, the extract of *P. sanctum* was the best because it presented a greater hypoglycemic effect at a lower dose (250 mg/kg of weight). When the dose was increased, the activity was lower: this may be due to the fact that regardless of the mechanism of the active principle, the effect depends on the concentration. However, the relationship of the effect with the concentration can be complex and is not usually linear [40]. This is the first report of hypoglycemic activity for this species of the *Piper genus*. It did not show toxicity on *A. salina*, unlike that reported by Déciga-Campos et al. [41] where it was significant.

The model of alloxan-induced diabetes has been widely used to evaluate the hypoglycemic activity of various plant extracts, in which a hypoglycemic effect has been reported within the first hours (1-8 h) after administration [42, 43]. This effect is also reported for drugs such as glibenclamide and tolbutamide, in these same periods postadministration. In the present study, we evaluated the hypoglycemic effect at 4 hours postadministration based on previous studies. This effect, when observed after the *β*-cells were destroyed, suggests that the extract could have a stimulating effect on the remaining *β*-cells, or we could suppose that the extracts favor the use of glucose at the cellular level with a similar effect. As for insulin, however, studies are needed to elucidate the possible mechanism of action [44–47].

## 4. Conclusions

Three plants evaluated showed hypoglycemic activity, *P. sanctum* being the one that showed the greatest reduction in glucose levels at a lower dose. This activity could be related to the presence of flavonoids and alkaloids that have already been associated with hypoglycemic activity. This plant did not present toxicity for *A. salina* at the doses evaluated, and this is the first time that it is reported to have hypoglycemic activity. The methanolic extract of *T. americana* var. mexicana presented antioxidant activity and had moderate toxicity. Further research is needed to identify the metabolites responsible for the biological activity of the plants evaluated.

## Figures and Tables

**Table 1:** Toxic activity on *A. salina* based on LC_50_ of each study extract. Values are expressed as mean ± SD.

Extracts	LC_50_ (ppm)	Category
*T. americana*	572.74 ± 5.72	Moderately toxic
*B. officinalis*	>1000	Nontoxic
*C. nuttalliae*	41.13 ± 3.12	Highly toxic
*P. sanctum*	>1000	Nontoxic

**Table 2:** DPPH radical scavenging activities of the extracts. Values are expressed as mean ± SD.

Extracts	EC_50_ (mg/mL)
*B. officinalis*	255.74 ± 5.24
*C. nuttalliae*	382.30 ± 8.32
*T. Americana* var. mexicana	8.84 ± 1.05
*P. sanctum*	482.30 ± 9.38
Tocopherol (positive control)	9.34 ± 2.02

## Data Availability

The data used to support the findings of this study are included within the article.
